# Physical inactivity before stroke is associated with dependency in basic activities of daily living 3 months after stroke

**DOI:** 10.3389/fneur.2023.1094232

**Published:** 2023-02-07

**Authors:** Jevgenijs Susts, Malin Reinholdsson, Katharina Stibrant Sunnerhagen, Tamar Abzhandadze

**Affiliations:** ^1^Department of Education and Science, National Rehabilitation Center “Vaivari”, Jurmala, Latvia; ^2^Faculty of Residency, Riga Stradins University, Riga, Latvia; ^3^Department of Clinical Neuroscience, Institute of Neuroscience and Physiology, University of Gothenburg, The Sahlgrenska Academy, Gothenburg, Sweden; ^4^Department of Occupational Therapy and Physiotherapy, Sahlgrenska University Hospital, Gothenburg, Sweden; ^5^Rehabilitation Medicine, Neurocare, Sahlgrenska University Hospital, Gothenburg, Sweden

**Keywords:** pre-stroke, physical activity, independence, assistance, functional outcome, exercise, sedentary behavior

## Abstract

**Background:**

Physical inactivity is a leading risk factor for non-communicable diseases, including stroke. Moreover, physical inactivity before stroke is associated with stroke severity, which, in turn, can cause disability. However, it remains unclear whether physical inactivity before stroke is associated with dependency in basic activities of daily living (ADL).

**Aim:**

The aim of this study was to evaluate whether physical inactivity before stroke influences ADL dependency 3 months after stroke.

**Methods:**

This longitudinal study was based on data from three Swedish registries. Patients with acute stroke who were admitted to the Sahlgrenska University Hospital between 9 November 2014 and 30 June 2019 were included in the study. Baseline data were collected from the three stroke units, and self-reported questionnaires were used to collect 3-month follow-up data. Physical inactivity before stroke was the primary independent variable that was self-reported using the Saltin–Grimby physical activity level scale. ADL dependency was a composite measure of three tasks: mobility, dressing, and toilet use. A binary logistic regression analysis was used to explain the association between physical inactivity before stroke and basic ADL 3 months after stroke.

**Results:**

In total, 3,472 patients were included in the study. The median age was 75 years, 49% of the patients were physically inactive before stroke, and 75% had a mild stroke. ADL dependency at follow-up was reported to be 32%. Physically inactive patients, compared with physically active patients, had 2.35 times higher odds for ADL dependency 3 months after stroke (odds ratio 2.30 [95% CI 1.89 – 2.80]). The model correctly classified 84% of the patients (the area under the receiver operating characteristic curve was 0.84 [95% CI, 0.83 – 0.86]).

**Conclusion:**

The findings of this study suggest that physical inactivity before stroke is associated with dependency in basic ADL 3 months after stroke. In addition, older age, female sex, pre-stroke living conditions, need for help, previous stroke, and admission stroke severity are significant contributors to dependency.

## 1. Introduction

Stroke is associated with a high burden on the healthcare system due to the loss of disability-adjusted life years (1, 2). Some level of assistance is required in 26–44% of stroke survivors (3, 4). By 2047, the number of stroke survivors in Europe is estimated to increase by 27% (5). After stroke, patients and their relatives turn to healthcare professionals for evaluating their recovery prognosis and assessing their need for assistance in activities of daily living (ADL) (2, 6, 7).

There are several modifiable and non-modifiable risk factors for stroke (8). Hypertension, hyperlipidemia, diabetes, smoking, physical inactivity, unhealthy diet, and obesity can increase stroke incidence (8). However, a few studies have explored whether these factors can influence functional outcomes. The risk factor of physical inactivity is estimated to be present among 31% of the global population, with continuously growing numbers (9). Physical inactivity is defined as not meeting the international recommendations of at least 150 min of moderate-intensity physical activity per week (10). Furthermore, there are conflicting results regarding how physical activity before stroke influences ADL after stroke (11, 12).

Studies have shown that additional factors, including older age, stroke severity, and ADL dependency before stroke, influence stroke-related functional outcomes (7, 13, 14). Moreover, men tend to have better functional outcomes, which may be related to a younger average age when experiencing their first stroke (15). Reperfusion therapy is associated with better outcomes in patients with moderate-to-severe ischemic stroke (16). Meanwhile, living alone is not associated with worse functional outcomes, although it is associated with higher mortality rates (17, 18).

Previous studies on physical inactivity before stroke have mostly looked at small samples and rarely evaluated how it influences dependency after stroke (12). While physical inactivity is acknowledged as a risk factor for stroke, it is also important to know whether it can influence functional outcomes after stroke. This study aimed to evaluate whether physical inactivity before stroke influences ADL dependence 3 months after stroke.

## 2. Materials and methods

### 2.1. Ethics statement

This study was approved by the Swedish Ethics Review Authority (^#^2021-03324, 13 July 2021). According to the Swedish Data Protection Authority, the handling of data generated within the framework of quality registries is exempt from the requirement for informed consent from participants. Furthermore, the Personal Data Act (Swedish Law ^#^1998:204, issued 29 April 1998) allows data from medical charts to be collected for clinical purposes and quality control without written-informed consent. Data collection and handling in this study followed the General Data Protection Regulation of Sweden (2018).

### 2.2. Study design and population

This longitudinal and registry-based study was a part of the research project Consequences After Stroke in Gothenburg (COASTGOT). Data were retrieved from three Swedish registries, including Väststroke, Riksstroke, and Statistics Sweden (SCB). Väststroke is a local quality registry for stroke with three stroke units at the Sahlgrenska University Hospital (SU) registering data. The catchment area for hospitals' basic care is 700,000 people, and specialized care is for 1.7 million people. Riksstroke is a national quality registry for stroke, covering over 90% of patients admitted to Swedish hospitals (19). SCB is Sweden's governmental, national statistics center.

This study included patients who were admitted to the SU between 1 November 2014 and 30 June 2019, were ≥18 years at the onset, had a stroke diagnosis (I61, non-traumatic intracerebral hemorrhage; I63, cerebral infarction; and I64, stroke not specified as hemorrhage or infarction), and had registered data on physical activity level before stroke and ADL 3 months after the stroke. Patients who died ≤93 days before follow-up were excluded from the analysis.

### 2.3. Procedure

Data from Väststroke, Riksstroke, and SCB were merged by statisticians at Riksstroke and SCB using the patients' personal identification numbers. Thereafter, personal identification numbers were replaced with serial numbers. SCB held the code key, and the researchers received a pseudonymized data file. The major reason for merging the data was the variety of variables available using several registries.

Data in Väststroke were registered by healthcare professionals working in the stroke units. Physiotherapists assessed and registered the level of physical activity during their first encounter with the patient. In addition, information was obtained from the next of kin when the patient could not respond, and medical doctors assessed stroke severity on hospital admission.

The baseline data on Riksstroke were registered by Riksstroke nurses working in the stroke units. Medical charts of patients were used as data sources. Three-month follow-up data, including information on basic ADL, were collected using self-reported postal questionnaires. In case of no response, a reminder letter was sent.

Data from the SCB were registered by governmental institutions. The authors retrieved information on the sociodemographic characteristics of patients and data on death after stroke. This study is reported in accordance with the STROBE guidelines for cohort studies (20).

### 2.4. Variables

The physical activity before stroke was assessed with Saltin–Grimby physical activity level scale (SGPALS) (21, 22). The SGPALS has four levels: (1) physically inactive, (2) light physical activity for at least 4 h/week, (3) moderate physical activity and training for at least 2–3 h/week, and (4) high-intensity physical training for competitive sports several times/week. The physical activity level refers to the year before the stroke. For statistical analysis, SGPALS was dichotomized into the physically inactive group including level 1 versus the physically active group with levels 2–4 (23). The SGPALS has good predictive validity (11, 22, 23).

The outcome variable was a composite measure with three basic ADLs: mobility, dressing, and toilet use. Mobility had three response categories that are as follows: (1) able to move around without help both indoors and outdoors (use of walking aid permitted), (2) able to move around without help indoors but not outdoors (use of walking aid permitted), and (3) need help from another person when moving around or bedridden. Levels 2 and 3 were considered dependent in mobility. Dressing and toilet use were binary variables describing “I need help” or “I can manage myself.” Dependency in basic ADL 3 months after stroke was defined as the need for help in at least one of the three basic ADLs (24).

The stroke severity was assessed during hospital admission using the National Institutes of Health Stroke Scale (NIHSS) (25). The score range of NIHSS is from 0 to 42 points, with a higher score indicating a more severe stroke. Stroke severity was stratified based on the NIHSS scores as follows: no neurological symptoms according to NIHSS (NIHSS 0 p), mild stroke (NIHSS 1–5 p), moderate stroke (NIHSS 6–14 p), and severe stroke (NIHSS ≥ 15 p).

Other variables included in the analysis were demographic characteristics (sex and age), ADLs (a composite measure of three basic ADLs before stroke), accommodation before the onset of stroke, comorbidities (previous stroke and diabetes), and reperfusion treatments. Notably, all variables were binary yes/no, except age, which was analyzed as a continuous variable. Accommodation before stroke was stratified into people who lived in their own homes without help, in their own homes with help, or in nursing homes (or equivalent) (19).

### 2.5. Data analysis

The difference between included and excluded patients was analyzed using the Mann–Whitney U-test for continuous variables and the chi-square test for categorical variables. The characteristics of the study sample are described as mean and standard deviation (SD), median and interquartile range (IQR), minimum–maximum, or numbers and frequencies (n [%]). The levels of physical activity before stroke were stratified into three groups for describing the characteristics of the study sample, and physical activity levels 3 and 4 were merged, as level 4 comprised only 11 observations. The statistical difference between the physical activity groups was studied with the Kruskal–Wallis test for independent continuous variables and the chi-square test for independent categorical variables.

#### 2.5.1. Choosing the regression model

A binary logistic regression analysis was performed to explain the dependency in basic ADL 3 months after stroke, as the outcome was a binary variable (ADL dependency defined as an event, “1”).

#### 2.5.2. Selection of explanatory variables

**Figure 1:**
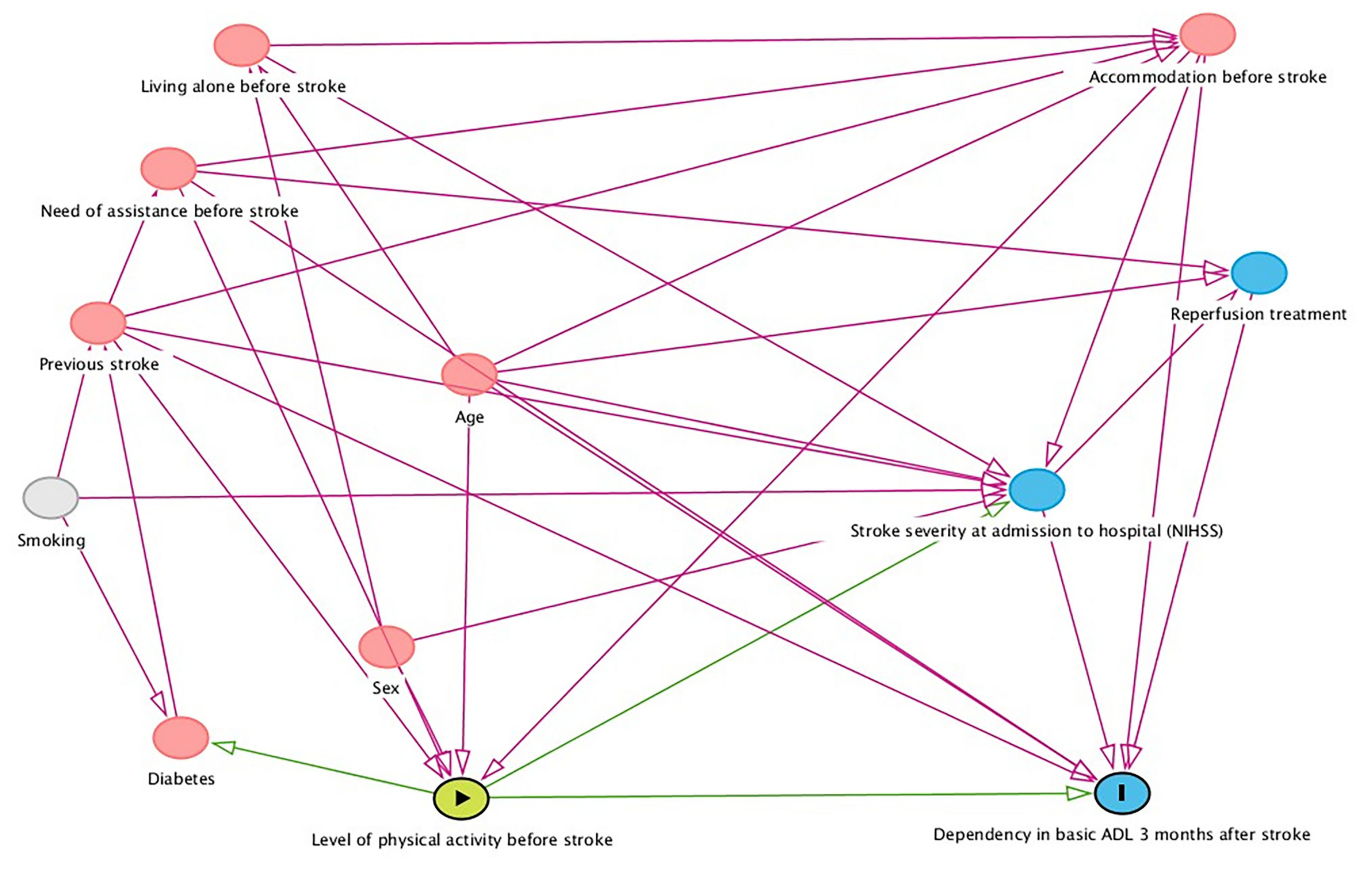
Directed acyclic graph showing factors that might confound the relationship between level of physical activity before stroke and dependency in basic activities of daily living (ADL) 3 months after stroke. NIHSS, National Institutes of Health Stroke Scale.

The primary explanatory variable was physical inactivity before stroke, with 10 potential independent variables. A directed acyclic graph (DAG) was created to make the regression model parsimonious and clinically relevant (Figure 1). The DAG model selected the five variables: age at stroke onset, accommodation before stroke, need for assistance before stroke, previous stroke, and stroke severity at admission to hospital for minimal adjustment variables to estimate the direct effect of physical inactivity before stroke on dependency in basic ADL 3 months after stroke. Although the DAG model did not select sex, it was still entered into the model as a variable regarded as clinically important (26).

#### 2.5.3. Fitting and evaluation of the regression models

All categorical explanatory variables were checked for ≥10 observations per outcome category. Multicollinearity between explanatory variables was studied by exploring the correlation coefficients and variance inflation factor (VIF). In the correlation analysis, the phi value was used to compare nominal variables, and Spearman's rho was used for continuous variables. A correlation of r <±0.7 was considered as not having multicollinearity (27). A VIF coefficient of less than two was regarded as acceptable.

The regression model was evaluated using the following tests: omnibus test (p < 0.05, good fit), Hosmer–Lemeshow test (*p* > 0.05, good fit), and Nagelkerke R^2^ test (a value closer to 1 was anticipated). The area under the receiver operating characteristic curve (AUC, a value closer to 1 was anticipated) was used to evaluate the model's ability to discriminate patients who were ADL-dependent from patients who were not. The binary logistic regression results at the variable level were evaluated with β coefficient, odds ratio (OR) with 95% confidence interval (CI), and a *p*-value. A sensitivity analysis was performed on the subgroup of patients who were fully independent before stroke.

Analyses were performed on a group of people who were physically inactive and physically active before stroke. The test variables were ADL dependence before stroke (no/yes) and 3 months after stroke (no/yes). Transition probabilities for each event were calculated. All analyses were performed using the SPSS software (IBM Corp. IBM SPSS Statistics for Windows, version 28.0. Armonk, NY). All statistical tests were two-sided at an alpha of 5%.

## 3. Results

**Figure 2:**
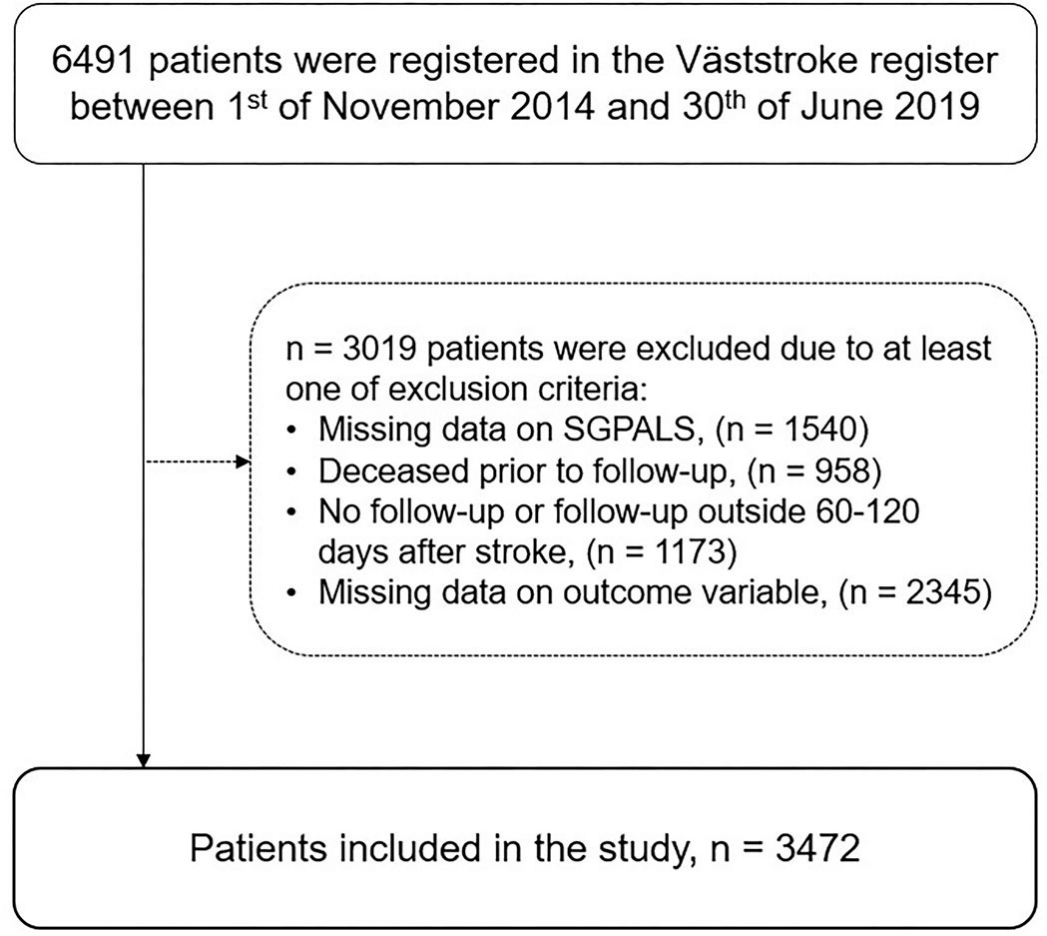
Flowchart of the study participants (SGPALS, Saltin–Grimby physical activity level scale).

In total, 3,472 patients were included in the study from the dataset that comprised 6,491 patients (Figure 2). Significant differences were found between included patients (*n* = 3,472) and excluded patients (*n* = 3,019) with more severe strokes on admission (median NIHSS score six points, *p* < 0.001), older age (median 77 years, *p* < 0.001), and more women (49%, *p* < 0.01) among the excluded patients.

Of 3,472 patients, 49% (*n* = 1,712) were physically inactive before stroke, 44 % (n = 1,521) reported light physical activity, and 7% (n = 239) reported moderate-/high-intensity physical activity and training (Table 1). Moreover, the median age of patients was 75 years, and 54% were men (Supplementary Table 1). ADL dependency before stroke was reported by 19% (*n* = 311) of the physically inactive group and 2% (*n* = 30) of the physically active group (*p* < 0.001). Patients who were dependent at the 3-month follow-up were older, with a higher proportion of physical inactivity, and had a more severe stroke than independent patients (Supplementary Table 1).

**Table 1:** Characteristics of the study sample stratified based on the levels of physical activity before stroke.

	**Levels of physical activity before stroke, SGPALS**			
**Characteristics**	**Physically inactive**	**Light physical activity**	**Moderate/high intensity physical activity and training**	***P*** **-value**
	***n** = **1,712***	***n** = **1,521***	***n** = **239***	
**Age, years**				<0.001^#^
Mean (SD)	76.1 (12.3)	71.6 (12.6)	61.5 (16.1)	
Median (IQR [min-max])	78 (16 [19–100])	73 (16 [20–99])	65 (20 [20–95])	
**Sex, n (%)**				<0.001
Male	810 (47)	891 (59)	177 (74)	
Female	902 (53)	630 (41)	62 (26)	
**Help in ADL or instrumental activities before stroke, n (%)**				<0.001
No	1,126 (71)	1,384 (95)	231 (100)	
Yes	471 (29)	75 (5)	1 (<1)	
**Accommodation before stroke, n (%)**				<0.001
Own accommodation without help	1,226 (72)	1,438 (95)	238 (100)	
Own accommodation with help	344 (20)	72 (5)	0 (0)	
Nursing home	*136 (8)*	10 (<1)	0 (0)	
Other	5 (<1)	0 (<1)	1 (<1)	
**Pre-morbid conditions**				
Independent in basic ADL before stroke, n (%)	1,352 (81)	1,480 (98)	239 (100)	<0.001
Living alone before stroke, no, n (%)	934 (55)	563 (37)	68 (29)	<0.001
Diabetes, yes, n (%)	383 (22)	233 (15)	8 (3)	<0.001
Previous stroke, yes, n (%)	320 (19)	184 (12)	14 (6)	<0.001
**Stroke type, n (%)**				0.44
Hemorrhagic stroke	150 (9)	140 (9)	16 (7)	
Ischemic stroke	1,562 (91)	1,381 (91)	223 (93)	
**Stroke severity at admission (NIHSS)**				<0.001^#^
Mean (SD)	4.7 (5.6)	3.5 (5.1)	3.0 (4.7)	
Median (IQR [min-max])	3 (6 [0–29])	1 (4 [0–28])	1 (4 [0–23])	
**Reperfusion treatment, n (%)**				<0.001
Yes	258 (16)	290 (21)	54 (24)	
No	1,343 (84)	1,117 (79)	173 (76)	

Statistics: ^#^Kruskal–Wallis test and chi-square test.

IQR, interquartile range; SD, standard deviation; NIHSS, National Institutes of Health Stroke Scale; SGPALS, Saltin–Grimby physical activity level scale; ADL, activities of daily living.

Variables with missing data n (%): previous stoke 9 (<1); stroke severity 176 (5); reperfusion treatment 237 (7); accommodation before stroke 2 (<1); living alone before stroke 29 (<1); need of assistance before stroke 184 (5); diabetes 4 (<1).

At the 3-month follow-up, dependency in basic ADL was reported by 32% (*n* = 1,119) of the patients, three times higher compared to that before stroke (*n* = 342, 10%). Patients who were dependent in basic ADL at follow-up were more often physically inactive before stroke (73%) than independent patients (38%).

The multivariable binary logistic regression analysis showed that physically inactive patients had 2.30 times higher odds for ADL dependency 3 months after stroke (OR 2.30 [95% CI 1.89–2.80]) than physically active patients. The variance of the model was 41% (Nagelkerke R square, 0.41). The regression model correctly classified 84% of the patients (AUC 0.84 [95% CI, 0.83–0.86]) as described in Table 2.

**Table 2:** Results of the multivariable binary logistic regression analysis for explaining dependency in basic activities of daily living 3 months after stroke.

**Explanatory variables**	**β (SE)**	**Adjusted *P*-value**	**Adjusted OR (95% CI)**
Physically inactive before stroke (SGPALS, level 1)	0.83 (0.10)	<0.001	2.30 (1.89–2.80)
Age (range 19–100 y)	0.06 (0.01)	<0.001	1.06 (1.05–1.07)
Female sex	0.28 (0.10)	0.008	1.33 (1.09–1.61)
***Ref***. Own accommodation without help			
Own accommodation with help	0.62 (0.17)	<0.001	1.87 (1.34–2.62)
Nursing home	1.28 (0.32)	<0.001	3.58 (1.92–6.68)
Living alone before stroke	0.26 (0.11)	0.81	1.03 (0.84–1.26)
Need help before stroke	0.91 (0.16)	<0.001	2.49 (1.83–3.39)
Stroke severity at admission to the hospital (NIHSS, range 0–29 p)	0.31 (0.01)	<0.001	1.14 (1.12–1.16)
Previous stroke	0.45 (0.14)	0.001	1.57 (1.19–2.05)

Statistics: binary logistic regression analysis. Predicted outcome: dependency in basic activities of daily living 3 months after stroke. Missing data, n = 351.

Model evaluation metrics: Hosmer–Lemeshow test, p = 0.10; Omnibus test for the model, p < 0.001; Nagelkerke R square, 0.41. The area under the receiver operating characteristic curve, 0.84 (95% CI, 0.83–0.86). SGPALS, Saltin–Grimby physical activity level scale; NIHSS, National Institutes of Health Stroke Scale; β, unstandardized regression coefficients; SE, standard error; OR, odds ratio; CI, confidence intervals.

The multivariable binary logistic regression analysis on the subgroup of the patients, who were independent before stroke, showed that physically inactive patients had 2.18 times higher odds for ADL dependency 3 months after stroke (OR 2.18 [95% CI 1.79–2.66]) than physically active patients. However, the variance and classification accuracy of the model were lower compared to the full model, 30 % (Nagelkerke R square, 0.30) and 80 % (AUC 0.80 [95% CI, 0.78–0.82]), respectively (Supplementary Table 2).

### 3.1. Subgroup analysis

**Figure 3:**
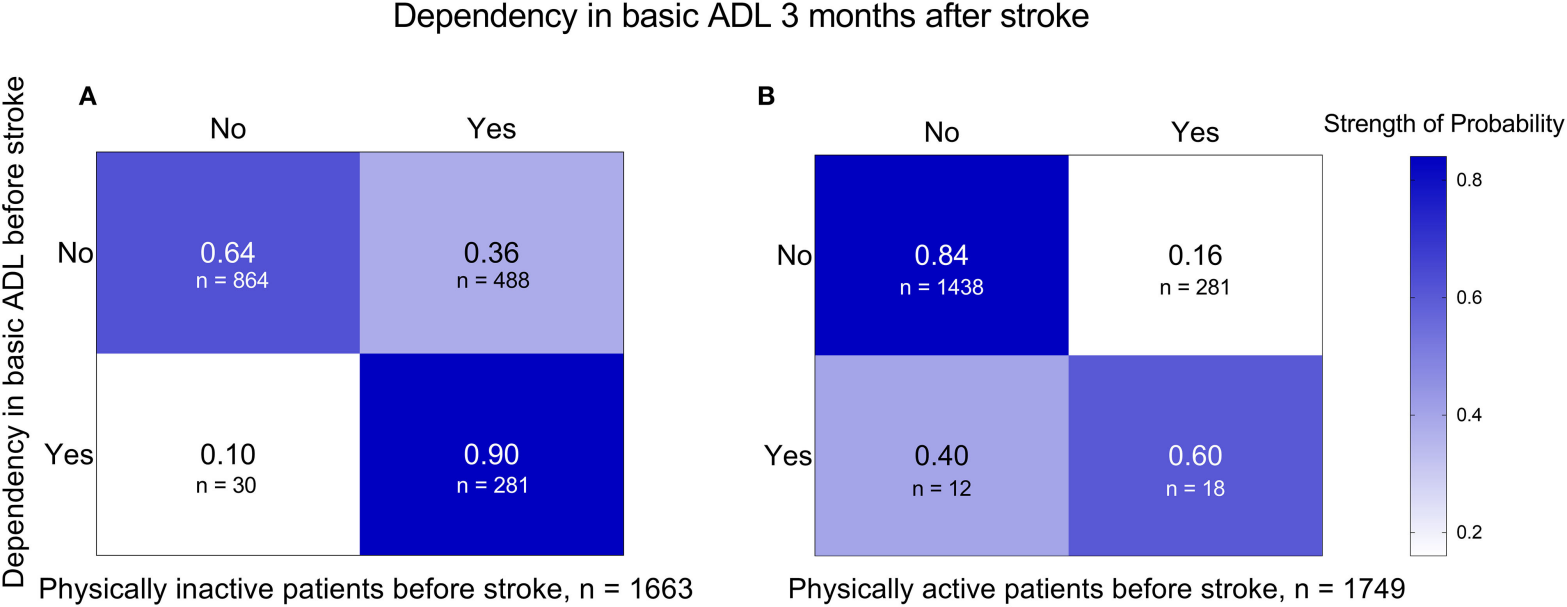
Transition probabilities from basic ADLs before stroke to basic ADLs 3 months after stroke in subgroups of patients who were physically inactive **(A)** and physically active **(B)** before stroke. ADL, activities of daily living.

The transition probability from ADL independence before stroke to ADL dependency 3 months after stroke was 0.36 and 0.16 in patients who were physically inactive and active before stroke, respectively (Figure 3).

**Figure 4:**
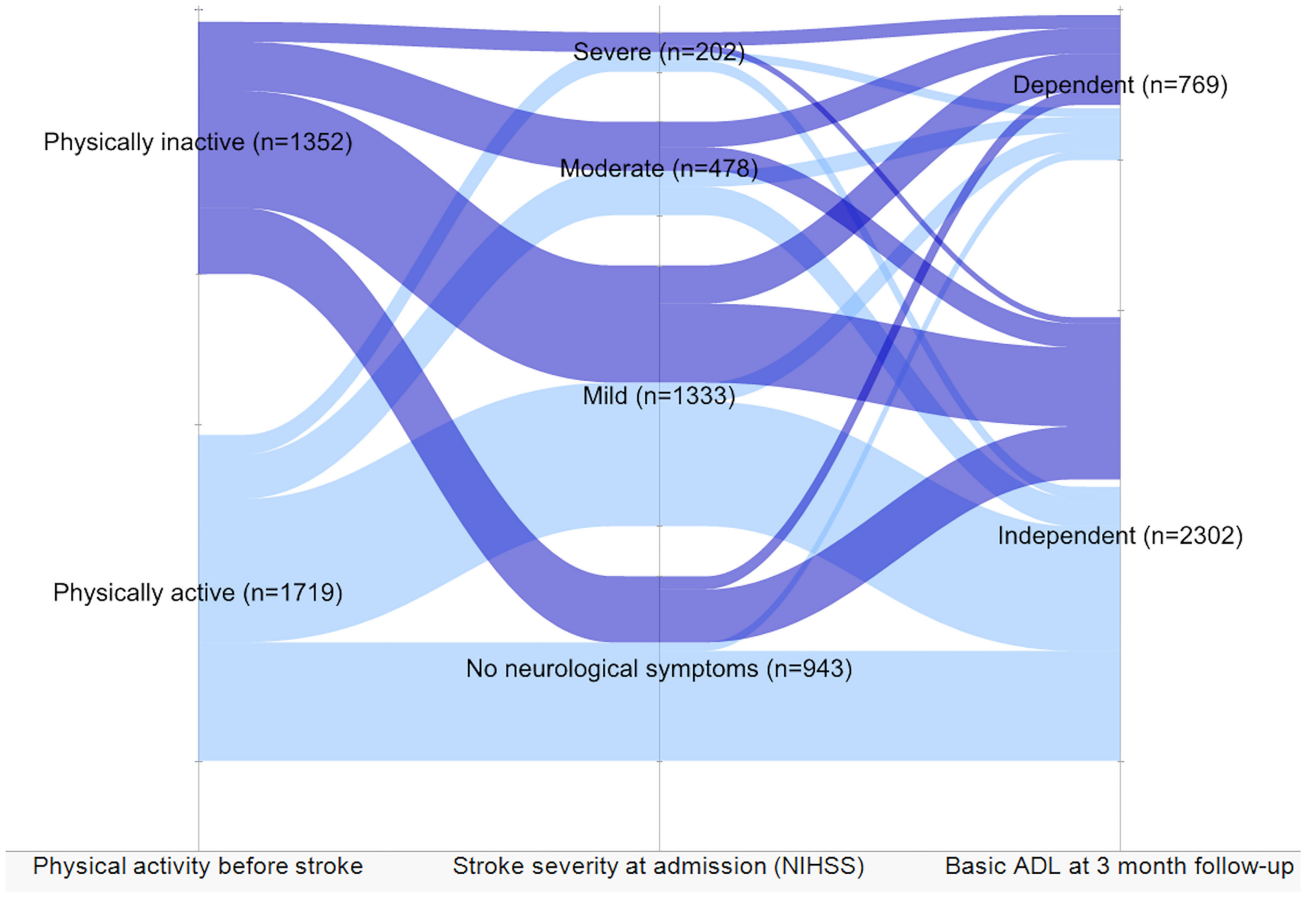
Level of dependency in activities of daily living (ADL) and stroke severity in patients independent before stroke based on the level of physical activity before stroke. The patients divided by physical activity level before stroke showed stroke severity and dependency 3 months after stroke.

Furthermore, a sensitivity analysis was performed on a group of patients with no prior ADL dependency. As shown in the Sankey diagram (Figure 4), despite stroke severity, physically inactive patients showed a trend toward dependency in basic ADLs 3 months after stroke.

## 4. Discussion

This study showed that patients who were physically inactive before stroke had higher odds of ADL dependency 3 months after stroke. In addition, we revealed that sociodemographic factors, pre-stroke living conditions, previous stroke, and admission stroke severity were significant factors. Physically active patients have a less severe stroke (28), which may be induced by a higher cardiovascular and neuromuscular reserve (29).

Moreover, physically inactive patients were often ADL-dependent 3 months after stroke, regardless of ADL ability before stroke. Although this study emphasizes on physical inactivity, the results support seven previous studies reviewed by Victorisson et al. where an association was found between a higher level of physical activity before stroke and less post-stroke disability (12). These findings support the hypothesis that physical inactivity before stroke is associated with ADL dependency after stroke. Nevertheless, four studies in this review reported no association (12). These conflicting results may be explained by different factors. First, the assessment scales for physical activity and ADL differed. Second, the timing of assessments varied. Third, the stroke cohorts differed in terms of their stroke characteristics.

In this study, other contributors to ADL dependency in addition to physical inactivity before stroke were sociodemographic factors, pre-stroke living conditions, previous stroke, and admission stroke severity. These results could be explained by the complexity of ADL ability considering that body functions and structures, as well as daily life engagement, can be impaired after stroke (7, 13, 30). Old age and high-stroke severity are well-known predictors of ADL dependency after stroke, confirming the results of our study (13). Moreover, female sex and previous stroke were associated with ADL dependency 3 and 12 months after stroke (24). Age and previous stroke were related to other factors in the regression model. Elderly patients and patients with comorbidities are more likely to live alone, in nursing homes, or need assistance in everyday life (31, 32). Unexpectedly, living alone before stroke was not associated with dependency 3 months after stroke. This result conflicts with a previous study that showed a positive association between living alone and ADL dependency 1 year after stroke (33).

This study has several strengths and limitations. The study was based on consecutively collected data from three stroke units. A large sample of patients had both ischemic and hemorrhagic stroke; the majority had mild strokes, and the median age was 75 years. Therefore, our sample can be assumed to be representative of the Swedish stroke population (26). However, a large number of participants were excluded from the analysis, mainly due to missing data on the primary explanatory variable and the outcome variable. This could lead to bias regarding the study sample. Therefore, the results should be interpreted with caution.

The study sample comprised patients with a wide range of pre- and post-stroke conditions and abilities. However, patients without a 3-month follow-up were older and had a more severe stroke, which could have influenced their ability to answer the questionnaires. Moreover, this group included a higher proportion of patients living in nursing homes and deceased patients.

The data were collected in clinical settings and represented a combination of patient-reported information and assessments performed by trained healthcare staff at stroke units. However, retrospectively collected data are associated with recall bias, particularly in populations with an increased risk of cognitive deficit. The next of kin was contacted by healthcare staff to reduce the risk of bias.

Physical activity before stroke was evaluated using the SGPALS. Although objective measurements are considered more accurate, they are not feasible for registry-based studies. The self-reported instruments are considered reliable and feasible for large samples (11, 29). The use of self-reported data to compare the results of different studies is problematic. In addition, the study outcome was a composite measured by three basic ADL questions, which were validated and are commonly used in Riksstroke-based studies (34). There are many assessment instruments that can be used for measuring the level of dependency after stroke; however, elaborating on these instruments can be difficult in a nationwide stroke registry that collects self-reported data.

## 5. Conclusion

The findings of this study suggest that physical inactivity before stroke is one factor associated with dependency in basic ADL 3 months after stroke. In addition, older age, female sex, pre-stroke living conditions, need for help, previous stroke, and admission stroke severity are also significant contributors to dependency. This study supports previous findings on the importance of physical activity in preventing the negative consequences of stroke. Therefore, promoting a physically active lifestyle could be a way to reduce the burden of stroke on society. However, these findings require additional knowledge. Future studies should investigate the levels of physical activity that are beneficial in decreasing stroke-related dependency. In addition to physical activity, it is important to investigate the influence of pre-stroke sedentary behavior on stroke outcomes.

## Data availability statement

The datasets presented in this article are not readily available because of ethical and privacy restrictions. Requests to access the datasets should be directed to KS, ks.sunnerhagen@neuro.gu.se.

## Ethics statement

This study was approved by the Swedish Ethics Review Authority (#2021-03324, July 13, 2021). Written informed consent for participation was not required for this study in accordance with the national legislation and the institutional requirements.

## Author contributions

JS: conceptualization of the study, manuscript drafting, data analysis, and interpretation of the results. MR: data acquisition, manuscript drafting, and interpretation of the results. KS: data acquisition, conceptualization of the study, and interpretation of results. TA: conceptualization of the study, data analysis, and interpretation of the results. All authors critically revised the manuscript for intellectual content and approved the final version.
