# Supplementary material for: Physical inactivity before stroke is associated with dependency in basic activities of daily living 3 months after stroke
Source: Front Neurol. 2023 Feb 7;14:1094232. doi: 10.3389/fneur.2023.1094232 (PMC9942155; doi:10.3389/fneur.2023.1094232)
Supplement: Supplementary file 1 [file Table_1.DOCX]

Online supplement

Supplementary Table 1. *Characteristics of the total sample and stratified based on basic ADLs three months after stroke*

|  |  | Basic ADL 3 months after stroke | | |
| --- | --- | --- | --- | --- |
| Characteristics | Total sample  (n = 3472) | Dependency  (n = 1119) | Independency (n = 2353) | |
| Age, years |  |  |  | |
| Mean (SD) | 73 (13.3) | 80 (108) | 70 (13.2) | |
| Median (IQR [min-max]) | 75 (17 [19–100]) | 82 (13[28–100]) | 72 (16[19–98]) | |
| Sex, n (%) |  |  |  | |
| Male | 1878 (54) | 474(42) | 1404 (60) | |
| Female | 1594 (46) | 645 (58) | 949 (40) | |
| Physical activity before stroke, SGPALS, n (%) |  |  |  | |
| Physically inactive (1) | 1712 (49) | 814 (73) | 898 (38) | |
| Light physical activity (2) | 1521 (44) | 289 (26) | 1232 (52) | |
| Moderate physical activity and training (3) | 228 (7) | 16 (1) | 212 (9) | |
| High intensity physical training (4) | 11 (<1) | 0 | 11 (<1) | |
| Living alone before stroke, no, n (%) | 1878 (54) | 467 (42) | 1411 (60) | |
| Accommodation before stroke, n (%) |  |  |  | |
| Own accommodation without help | 2902 (84) | 697 (63) | 2205 (94) | |
| Own accommodation with help | 416 (12) | 296 (26) | 120 (29) | |
| Nursing home | 146 (4) | 122 (11) | 24 (1) | |
| Other | 6 (<1) | 3 (<1) | 3 (<1) | |
| Independent in basic ADL before stroke, n (%) | 3071(90) | 769 (72) | 2302 (98) | |
| Help in ADL or instrumental activities before stroke, n (%) |  |  |  | |
| No | 2714 (84) | 627(61) | 2114(94) | |
| Yes | 547 (17) | 406(39) | 141(6) | |
| Diabetes, yes, n (%) | 624 (18) | 247 (22) | 377 (16) | |
| Previous stroke, yes, n (%) | 518 (15) | 252 (23) | 266 (11) | |
| Stroke type  Ischemic stroke  Hemorrhagic stroke | 3166 (91)  306 (9) | 984 (88)  135 (12) | 2182 (93)  171 (7) | |
| Stroke severity at admission (NIHSS) |  |  |  | |
| Mean (SD) | 4 (5) | 7 (6) | 3 (4) | |
| Median (IQR [min-max]) | 2 (6 [0–29]) | 5 (8 [0–29]) | 1 (4 [0–28]) | |
| Stoke severity stratified on HIHSS score, n (%) |  |  |  | |
| No neurological symptoms (NIHSS 0) | 991 (30) | 145 (14) | 846 (37) | |
| Mild stroke (NIHSS 1-5) | 1478 (45) | 412 (40) | 1066 (47) | |
| Moderate stroke (NIHSS 6-14) | 586 (18) | 314 (31) | 272 (12) | |
| Severe stroke (NIHSS ≥15) | 241 (7) | 148 (15) | 93 (4) | |
| Reperfusion treatment, n (%) |  |  |  | |
| Yes | 602 (19) | 190 (19) | 412 (19) | |
| No | 2633 (81) | 832 (81) | 1801 (81) | |
| Abbreviations: IQR, Interquartile Range ; SD, standard deviation; NIHSS, National Institutes of Health Stroke Scale; SGPALS, Saltin-Grimby Physical Activity Level Scale; ADL, Activities of daily living.  Variables with missing data n (%): previous stoke 9 (<1); stroke severity 176 (5); reperfusion treatment 237 (7); accommodation before stroke 2 (<1); living alone before stroke 29 (<1); need of assistance before stroke 184 (5): diabetes 4 (<1). | | | |  |

Supplementary Table 2.  *Results of the multivariable binary logistic regression analyses for explaining dependency in basic activities of daily living 3 months after stroke. Subgroup of patients who were fully independent before stroke, n = 3071.*

| Explanatory variables | β (SE) | Adjusted  P-value | Adjusted  OR (95% CI) |
| --- | --- | --- | --- |
| Physically inactive before stroke (SGPALS, level 1) | 0.78 (0.10) | <0.001 | 2.18 (1.79–2.66) |
| Age (range 19 - 100 y) | 0.06 (0.01) | <0.001 | 1.06 (1.05–1.07) |
| Female sex | 0.27 (0.10) | 0.007 | 1.32 (1.08–1.61) |
| ***Ref.*** Own accommodation without help |  |  |  |
| Own accommodation with help | 0.74 (0.17) | <0.001 | 2.10 (1.51–2.92) |
| Nursing home | 0.83 (0.47) | 0.077 | 2.30 (0.91–5.77) |
| Living alone before stroke | 0.07 (0.11) | 0.521 | 1.07 (0.87–1.32) |
| Stroke severity at admission to the hospital (NIHSS, range 0 - 28 p) | 0.13 (0.01) | <0.001 | 1.14 (1.12–1.16) |
| Having previous stroke | 0.42 (0.15) | 0.003 | 1.53 (1.15–2.03) |
| Statistics: Binary logistic regression analyses. Predicted outcome: dependency in basic activities of daily living three months after stroke. Missing data, n=143.  Model evaluation metrics: Hosmer and Lemeshow test, p = 0.04; Omnibus test for the model, p <0.001; Nagelkerke R square, 0.30. The area under the Receiver operating characteristic curve, 0.80 (95% CI, 0.78 – 0.82). Abbreviations: SGPALS, Saltin-Grimby Physical Activity Level Scale; NIHSS National Institutes of Health Stroke Scale; β, unstandardized regression coefficients; SE, standard error; OR, Odds Ratio; CI, Confidence intervals. | | | |
